# The Work of Radium Institutes

**Published:** 1915-03-06

**Authors:** 


### THE WORK OF RADIUM INSTITUTES.

### The Cases Received and the Results of Treatment.

The report of the work of the Radium Institute during 1914 is a document of much interest and importance. The number of cases—841—brought under observation, and the special experience of the medical officers, combine to give an assurance that any conclusions suggested rest on a very secure basis. From the special department of the Royal Infirmary, Edinburgh, there comes, too, a report on radium treatment, and though here the patients numbered only sixty-three, the record has a note of excellence which demands recognition. The results in cases of malignant disease hardly announce any new departure, but they confirm what has been announced in previous records; and in individual cases changes of a very striking character are registered. The most marked examples in this department continue to be found in cases of rodent ulcer, the large majority of which are noted either as "apparently cured" or "improved." In no single instance of malignant growth is a "cure" claimed, but the term "apparently cured" means that all traces of the lesion have disappeared, and that the patient is free from any indication or symptom of the disease. This, at least, is the rule at the Radium Institute, but the Edinburgh report is a little more confident, for it states that of twelve cases of rodent ulcer, nine were easily "cured," the remaining three having been under treatment too recently to permit a definite verdict. The question of dosage is evidently being recognised as of very great moment, and the Institute gives very precise details what amount of radium, what length of exposure, and what frequency of repetition are necessary in dealing with the various malignant types.

Turning now to individual groups of cases as classified at the Radium Institute, we may briefly summarise the recorded experience. In uterine cancer the results are said to be "most gratifying" and far in advance of any other known medical or surgical methods; this, of course, referring to inoperable cases, for cases suitable for operation are not accepted. Not only may there be disappearance of the fungating growth and cessation of pain, hæmorrhage, and discharge, but in favourable instances there seems to be a retarding influence in the dissemination of deposits and an arrest of the progress of the disease. Of six cases of carcinoma of the bladder "gratifying" results were noted in two instances.

Naturally the most numerous of the malignant cases were those of cancer of the breast, and here, speaking generally, the records are not very cheering. Of a total of eighty-seven cases treated, forty are noted as "improved" and only one as "apparently cured." It is recognised, too, that in long-continued cases there comes a stage at which the response to radium fails and the benefit derived becomes negligible.

Again, thirty-eight cases of carcinoma of the rectum were treated, and of these none appears in the "apparently cured" list, but twenty-two are marked "improved." These qualified results by no means imply that individual sufferers have not received much relief and benefit from the radium treatment, and indeed it is obvious from the report that the very opposite is the case, but they do suggest that too sanguine an outlook must not be cultivated. At the same time it must be remembered that the cases dealt with are almost invariably of an unpromising type and represent conditions which are beyond the reach of the surgeon. One very distinct example of benefit short of cure is quoted among the rectal cases, where we are told that in two or three instances growths which had been considered inoperable previous to the use of radium were so much diminished by the treatment that their removal has since been successfully accomplished. What has been said of frankly malignant cases applies also to such conditions as lymphadenoma and leukæmia. The improvement in the local conditions is conspicuous, but in the majority of cases recrudescence occurs, though now and again the disease remains quiescent for some time, occasionally for as long as a year.

Of great interest is the section dealing with non-malignant diseases, and particularly with chronic disease of the joints and with exophthalmic goitre. Of 206 cases of arthritis deformans treated at the London Institute ninety-one were reported as "improved." The method employed was the daily administration of 250 c.c. of radium emanation solution, and the remark is made that much perseverance is necessary and that at least six weeks are likely to elapse before any change can be observed. In certain cases "remarkable results" are recorded, and, so far as can be judged, the most promising are patients under forty years of age in whom the disease has a relatively short duration and is peri-articular in type and multi-articular in distribution. In some cases of this order not only was pain lessened, but a very considerable degree of mobility was regained in joints which had previously been fixed by fibrous thickenings. Considering the very obstinate and usually progressive character of arthritis deformans, these results are decided gains and encourage the hope of still better things in the future. It would be of interest to hear whether they could be confirmed at such an institution, say, as the Research Hospital at Cambridge.

Among the Edinburgh cases are included six cases of exophthalmic goitre, all treated, we are told, with "undoubted benefit," and in one case "a definite cure" was obtained; diminution in the exophthalmos and the tachycardia were amongst the earliest results to be observed.

It is rather surprising, in view of certain advertising schemes, to notice that arterio-sclerosis and high blood pressure have not come under review. One such case reported as "improved" is noted in the London report, but there the record ends. Skill and patience are being applied to radium therapeutics, and certain substantial benefits can be counted on.

